# Autonomous System for Lake Ice Monitoring

**DOI:** 10.3390/s21248505

**Published:** 2021-12-20

**Authors:** Ilya Aslamov, Georgiy Kirillin, Mikhail Makarov, Konstantin Kucher, Ruslan Gnatovsky, Nikolay Granin

**Affiliations:** 1 Department of Hydrology and Hydrophysics, Limnological Institute, Siberian Branch of Russian Academy of Sciences, 664033 Irkutsk, Russia; mmmsoft@hlserver.lin.irk.ru (M.M.); kost@hlserver.lin.irk.ru (K.K.); gnat@lin.irk.ru (R.G.); nick@lin.irk.ru (N.G.); 2 Deaprtment of Ecohydrology and Biogeochemistry, Lebiniz-Institute of Freshwater Ecology and Inland Fisheries (IGB), 12587 Berlin, Germany; kirillin@igb-berlin.de

**Keywords:** ice thickness, temperature, snow depth, monitoring, in situ measurements, instruments, Lake Baikal, heat fluxes

## Abstract

Continuous monitoring of ice cover belongs to the key tasks of modern climate research, providing up-to-date information on climate change in cold regions. While a strong advance in ice monitoring worldwide has been provided by the recent development of remote sensing methods, quantification of seasonal ice cover is impossible without on-site autonomous measurements of the mass and heat budget. In the present study, we propose an autonomous monitoring system for continuous in situ measuring of vertical temperature distribution in the near-ice air, the ice strata and the under-ice water layer for several months with simultaneous records of solar radiation incoming at the lake surface and passing through the snow and ice covers as well as snow and ice thicknesses. The use of modern miniature analog and digital sensors made it possible to make a compact, energy efficient measurement system with high precision and spatial resolution and characterized by easy deployment and transportation. In particular, the high resolution of the ice thickness probe of 0.05 mm allows to resolve the fine-scale processes occurring in low-flow environments, such as freshwater lakes. Several systems were tested in numerous studies in Lake Baikal and demonstrated a high reliability in deriving the ice heat balance components during ice-covered periods.

## 1. Introduction

Recently, the interest in the ice season has been remarkably increasing among limnologists [1,2], caused by the question of the response of mid- and high-latitude lakes to global warming. The remarkable development of measurement techniques in recent years ensures an additional impulse to intensify research on ice-covered lakes. Increased sensor accuracy and stability, as well as a higher memory capacity of internal loggers at lower power consumption, provide continuous self-contained recording of crucial physical characteristics such as solar radiation, temperature, conductivity and oxygen content with high accuracy and high temporal resolution. Consequently, it has become possible to obtain a qualitatively new, deeper insight into the physical nature of ice-covered lakes where transport processes are driven by small gradients in the background fields.

Seasonal formation of ice is an essential feature of the hydrological regime in temperate and (sub-)polar climatic zones. In this regard, freshwater lakes are a special class of hydrological objects both in terms of thermal and hydrodynamic processes that control the formation and melting of ice and from the point of view of the impact of the ice regime on the global freshwater budget. Seasonally freezing lake systems—Lake Baikal, Laurentian Great Lakes, great European Lakes Ladoga and Onego and lake systems of Fennoscandia and Northern Canada—accumulate the bulk of the world’s surface freshwater. Their seasonal ice regime defines the climatic balance of precipitation and evaporation as well as the ecosystem state and the water quality of the lakes themselves [3,4]. A steady trend of shortening of the ice season on lakes during the past 100 to 150 years was reported in numerous recent studies [5,6,7,8,9] and was attributed to global climate warming. Estimation of the consequences of these phenological changes on water resources requires the quantification of the physical mechanisms that control the formation and melting of ice. The heat and mass transfer at the ice–water interface is the least studied among these mechanisms. This is due to the difficulties in measuring the heat flux and its dependence on a number of physical processes, including absorption of solar radiation, temperature variability within the ice cover and in the under-ice water layer as well as the intensity of currents and turbulence in the water column. With this connection, a need has arisen for an instrument providing *in situ* autonomous continuous monitoring of vertical temperature distribution in the near-ice air, the ice strata and the under-ice water layer for several months with simultaneous registration of solar radiation incoming at the lake surface and passing through the snow and ice covers, under-ice current velocities and snow and ice thicknesses.

The first autonomous stations aimed at monitoring of ice properties were the so-called ice mass balance (IMB) buoys designed for marine conditions, among which there were the Cold Regions Research and Engineering Laboratory (CRREL) IMB buoy [10] and the Scottish Association for Marine Science (SAMS) IMB buoy [11]. They are relatively large, consist of several parts connected by wires and are difficult to deploy and retrieve from the ice. Furthermore, data controller housing and other parts were non-floating and relied on the ice for mechanical support, limiting their use for collecting data in seasonal ice. These limitations were addressed in 2011 with the advent of the Seasonal Ice Mass Balance buoy (SIMB-1), whose sensors were enclosed in one cylindrical upward-floating buoy hull [12]. Its further modifications led to the creation of the SIMB-3 buoy with higher reliability and maximized survivability as well as reduced instrument size, weight and cost [13]. The SIMB-3 is equipped with acoustic rangefinders installed in the air and under the ice base. The distances are measured between the downward-looking echo-sounder and the snow surface, and between the upward-looking echo sounder and the ice bottom, recalculated later to snow depth and ice thickness. CRREL buoys have been intensively used in the Arctic Ocean [14,15,16].

Modernization of the SAMS IMB buoy resulted in the Snow Ice Mass Balance Array (SIMBA) buoy, which is currently widely used, in particular because of its cost-cutting design. The buoy does not have any built-in rangefinders, but possesses a dense spatial distribution of temperature sensors in its thermistor string, spaced every 2 cm. Additionally, small heaters are mounted underneath every temperature sensor, emitting heat impulses once per day for 60–120 s. The different heating rates of the sensors in the various environments help to distinguish the air/snow, air/ice and ice/water interfaces. Despite this approach, retrieval of snow depth and ice thickness from the SIMBA temperature profiles remains a non-trivial task requiring manual data processing, while several automated algorithms of snow and ice depth determination were recently proposed [17,18]. Most SIMBA buoys were deployed in polar seas [19,20], and lake observations have also been reported recently [21].

The Polar Research Institute of China and Taiyuan University of Technology have developed their own ice-tethered buoys. This type of buoy is similar to SIMBA, and has a 4.5 m long thermistor string consisting of 150 sensors spaced every 3 cm. The buoys also include acoustic rangefinders in air and water [18]. The buoys have been widely deployed in the Arctic Ocean since the Chinese National Arctic Research Expedition (CHINARE-2014) [19,20].

Despite the advances in automated ice monitoring, all of these buoys provide relatively rough estimates of heat and mass balance. The temperature sensors used have a resolution of 0.0625 °C and initial accuracy of ±0.5 °C (up to ±0.1 °C after calibration), which are insufficient to resolve the fine-scale processes occurring at the ice–water interface and beneath it where small vertical temperature gradients are crucial for the melting rate at the ice base. Moreover, the massive body of the SIMB-3 buoy (12 cm in diameter) has a significant impact on the surrounding temperatures. The underwater rangefinder used in the SIMB-3 also has a resolution of only 1 cm.

The developers of the “Multilayer Sea Ice Temperature Sensor” [22] addressed these issues by using analog RTD temperature sensors with a complex circuit of amplification, multiplexing and digitizing of signals. They paid much attention to increasing the accuracy of temperature measurements by investigating the temperature dependence of the constant current sources, the amplification circuit and the analog-to-digital converter, and eventually achieved an accuracy of about ±0.005 °C. The accuracy improvements were, however, compromised by soldering the sensors on a single PCB, with a rather large final size after sealing (cross-sectional dimension 30 × 7.5 mm) and the use of a bulky support device for the sensor chain, which introduced additional perturbations to the measured temperature. Ding and Mao [23] designed an ice temperature measurement system based on analog RTD thermistors with a high accuracy of ±0.05 °C, but the linear dimensions of the system were relatively large. The standard CR1000X data logger was used to measure and transmit the temperature readings. Ice thickness was measured using a stave sliding through a hole made with an ice auger [23]. In addition, the sampling frequency of all the above devices is usually once per hour or less, which is often insufficient for a detailed description of temperature variability under the ice.

The prototype ice monitoring system of Aslamov et al. [24] has been specifically designed for monitoring lake ice where fluxes are typically lower than in marine systems. We used digital temperature sensors similar to that of SIMB-3 and SIMBA. Their resolution was found to be insufficient for reliable registration of the ice heat budget. Afterwards, the design underwent continuous changes. In 2010, we made a thermistor chain completely based on analog temperature sensors [25]. Later, the measurement system combined high-precision digital temperature sensors and fast-response analog sensors while retaining an integral one-piece design [26]. In the present study, we present the latest version of a monitoring system of heat and mass fluxes in the air–ice–water system designed for long-term autonomous deployment in the ice cover and recording of vertical fluxes with resolution and accuracy sufficient to overcome the abovementioned challenges and to estimate the heat budget of the seasonal ice cover in low-flow environments such as freshwater lakes. The system was tested in Lake Baikal, Russia and demonstrated high reliability in deriving the ice heat balance components during ice-covered periods.

## 2. Design of the Autonomous System for Lake Ice Monitoring

### 2.1. General Description of the System

The autonomous system for lake ice monitoring (ASLIM) is designed to record the vertical temperature distribution in the air, within the ice cover and sub-ice water layer together with short-wave solar radiation entering and passing through the snow and ice as well as the thickness of snow and ice. The 32-bit microcontroller PIC33EP256MU806 (Microchip Technology Inc., Chandler, AZ, USA), a link between all elements of the system, determines the logic of the entire device.

Ultra-low noise (4 μVRMS) low-dropout linear regulators based on TPS7A4700 (Texas Instruments, Dallas, TX, USA) chips are used to form supply voltages of 5 V and 3.3 V for measuring sensors. The device has a SIM900-based GSM module (SIMCom Wireless Solutions, Shanghai, China) for transmitting data to the external server via the Internet. The real-time clock module in the device is based on the DS3232MZ chip (Maxim Integrated) with a built-in high-stability temperature-compensated crystal oscillator having a redundant power supply. The internal clock is corrected by periodic time synchronization with an Internet server during data uploads. As an option, it is possible to install a GPS receiver for more accurate time synchronization via satellites. Readings from all sensors are recorded at configurable intervals of 1 minute or more and are stored on a removable micro-SD memory card. The FRAM FM24CL04 chip with an unlimited number of rewrite cycles is used to store the device settings and its status. The ASLIM can be powered by 4, 8 or 12 lithium thionyl chloride (Li-SOCl${}_{2}$) batteries, ER34615, providing continuous operation of the device for 3 to 12 months, depending on the configuration.

**Figure 1:**
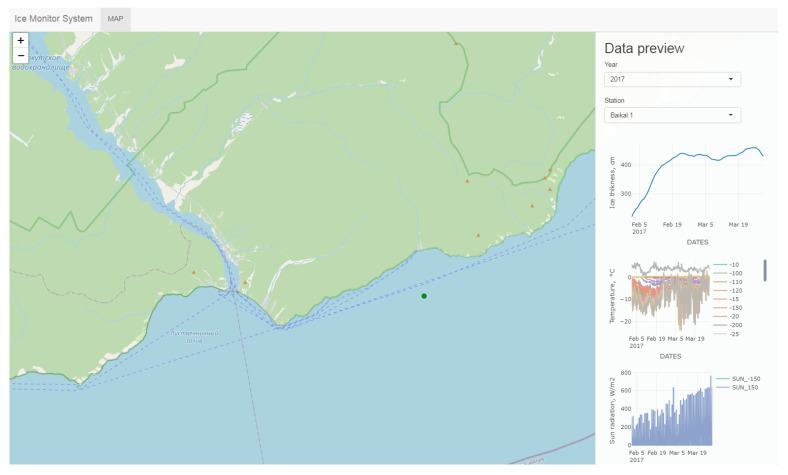
An example of a webpage of the server with a preview of the dataset from one of the experiments.

The information collected by the ASLIM is transmitted via a GSM network to a remote Internet server with configurable periodicity, typically every hour. The server hosts a database and a dynamic webpage for online visualization of information from the network of ice measuring systems (https://hlserver.lin.irk.ru/shs/icemon/ (accessed on 16 December 2021)). It is also possible to download raw data in tabular format for further processing and analysis after authorization. An example of a webpage with previewed data is shown in Figure 1. The “Data explorer” tab can be used for a detailed view of data graphs with zoom and pan features.

The design of the measurement part of the ASLIM was focused on the creation of a modular architecture providing a variety of sensor configurations built upon a single four-wire bus. The interface I${}^{2}$C was chosen as a physical layer protocol to ensure the exchange of information between the controlling device and the sensors. To ensure the modular structure, the measuring system was divided into 40 cm long sections. Each section has a set of sensors and a 16-bit PIC24F08KL200 microcontroller (Microchip Technology Inc., Chandler, AZ, USA) for operating the sampling. Each module is equipped with an I${}^{2}$C bus repeater based on the TCA9517 chip at the input, which significantly increases the possible total length of the measurement system while avoiding the effects of increased parasitic capacitance. The microcontroller supplies power to the sensors only when they are polled, thus reducing the overall ASLIM power consumption. A “bootloader” program was developed for the microcontroller, allowing its firmware to be updated at any time via the I${}^{2}$C bus after it has been sealed into the polyurethane. The miniature sealed connectors were cast using specifically designed molds providing, on one hand, easy interconnection of individual modules and, on the other hand, compactness of the ASLIM for transportation.

**Figure 2:**
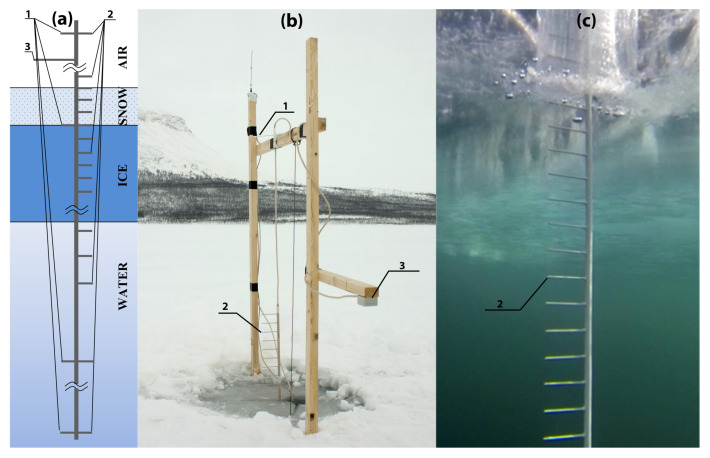
(**a**) Measuring system of the ASLIM: 1—light sensors, 2—temperature sensors, 3—snow thickness sensor; (**b**,**c**) photos of above-water and underwater parts of the measuring system, respectively.

The core measurement unit of the station consisted of a 5 m long "chain" of sensors frozen into the lake ice and measuring the continuous temperature profile in the air, ice and water with 5 cm resolution (Figure 2). The distance between sensors was increased to 10–50 cm in the water layer deeper than one meter beneath the ice base. The system included four light sensors: one sensor is located 1.5 m above the ice surface in the air, another at the top of the ice, and the remaining two 1.5 m and 2.5 m beneath the ice surface in the water. An ultrasonic snow cover thickness meter with spatial resolution of 1 mm and an accuracy of 1 cm is placed in the air, at a distance of 1 m from the ice surface. The station is also equipped with an ultrasonic range finder deployed in water and measuring the variations in the ice bottom position with 0.05 mm resolution and 0.2 mm accuracy. All components of the system were made in white to minimize the effects of heating by solar radiation.

To install the autonomous system, a hole 130 mm in diameter is drilled in the ice (slightly away from the support system), through which the logger body is immersed. Two cuts from the hole at right angles are made with the help of an ice saw. In one cut the measurement system is inserted, and positioned so that the second light sensor from the top and the corresponding temperature sensor are aligned with the upper ice surface (Figure 2a,b). The sensors are buried down to several millimeters under the surface and covered with water during the installation to ensure that they are frozen and measure the temperature of the ice surface rather than that of the surrounding air. Through the second cut the logger hanging tether is moved aside, so that the acoustic rangefinder receives reflection from the undisturbed surface of the ice cover. Finally, the measurement system and hanging tether are fixed on a crossbar (Figure 2b). Thin cuts filled with water at about 0 °C freeze very quickly, allowing measurement of the actual temperature distribution in the ice on the day after installation.

### 2.2. Temperature Sensors

To measure the ambient temperature, two types of sensors were used in the instrument.

**Figure 3:**
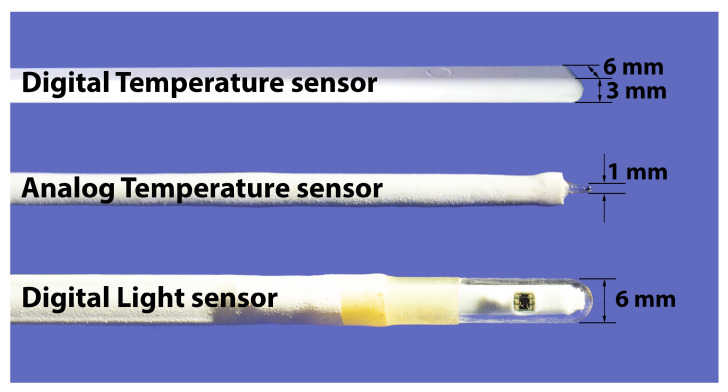
Temperature and light sensors.

MAX31725 chips were chosen as the main temperature sensors. The chip is a miniature temperature sensor based on a P-N junction with integrated on-chip ADC, a digital core and I${}^{2}$C interface. MAX31725 allows up to 32 instances on a single I${}^{2}$C bus. The on-chip 16-bit ADC enables high-resolution temperature measurements of 0.004 °C every 50 ms. By averaging the counts for two seconds, the resolution is increased up to <0.001 °C. The microcontroller supplies power to the temperature sensors only when they are polled, thus reducing the overall power consumption. Each temperature sensor is mounted on a separate board that is soldered into the main board at a right angle with a 10 cm distance between the sensor tip and the main board of the measurement system (Figure 2), minimizing the temperature influence of the main support rod (which is 14 mm in diameter) on the temperature records. The small (0.8 mm thick and 3 mm thick with PCB after sealing) sensors provide measurements of the temperature profile with high vertical resolution (Figure 3).

Additionally, fast-response analog temperature sensors were used in the range of the possible location of the ice–water interface to obtain higher resolution in temperature and space. Glass sealed semiconductor thermistors were used as sensing elements (Figure 3) with 1 mm diameter, with response time of <1 s and nominal resistance of 2.2 kOhm. To achieve the best results in terms of accuracy and stability of the temperature measurements, a ratiometric scheme was applied. Three temperature sensors were connected in series with a 10 kOhm Y162410K0000T9R reference resistor (Vishay precision group, Malvern, PA, USA), which is an ultra-high-precision foil wraparound chip resistor with ±0.01% tolerance and extremely low TCR of only 0.05 ppm/°C. As the same current flows through every part of the circuit, this design ensures that the analog input voltage remains ratiometric to the reference voltage, and any errors in the analog input voltage due to the temperature drift and noise of the excitation current are compensated by the variation in the reference voltage drawn from the high-stability resistor. The four-wire connection of each sensor was utilized, canceling the error due to lead resistance. An ADS1247 analog-to-digital converter (ADC) (Texas Instruments, Dallas, TX, USA) was used to accurately measure the voltage from each of the three thermistors. This is a miniature precision four-channel 24-bit ΔΣ ADC that includes a low-noise programmable gain amplifier, internal oscillator, low-drift voltage reference and two built-in programmable excitation current sources.

The device has an adjustable burst mode for recording the readings of all low-inertia analog temperature sensors with a 1 Hz frequency. The data on high frequency and fine-scale temperature fluctuations can provide information on under-ice convection and the characteristics of turbulent mixing in the ice–water boundary layer as well as evaluate the dissipation rate of the turbulent kinetic energy (TKE) [27].

### 2.3. Light Sensors

The highly integrated optical sensors, MAX44006 and MAX44008 (Maxim integrated, San Jose, CA, USA), were used to measure downward planar irradiance. These chips combine five types of high-sensitivity (down to 0.001 Lux) photodiodes, which can measure intensity in the red, green, blue and infrared spectrum (maximum sensitivity at 630, 538, 470 and 850 nm, respectively) and also intensity of ambient light (with identical sensitivity to the photopic curve of the human eye). A built-in temperature sensor is used to compensate for the influence of temperature changes on the illumination and infrared sensor readings. Six on-chip ADCs enable simultaneous measurement of signals in all channels. The sensor outputs via digital interface values calibrated in W/m${}^{2}$. A miniature glass sheath on the sensor protects it from water and, at the same time, serves as a cosine collector (Figure 3). The other side of the sensor is sealed with polyurethane, and silica gel balls are placed inside to prevent possible fogging. Light sensors are mounted on a separate board soldered into the main board at a right angle, ensuring that the sensor is 10 cm away from the main rod in the opposite direction to the temperature sensors. During the installation of the ASLIM, the light sensors are directed towards the south to prevent them from being obscured by other parts of the measuring system.

The presence of light sensors in the air, ice and under-ice water allows for estimating the attenuation of solar radiation in the ice and the water column, whereas the use of information on the thickness of the snow enables studying its effect on the amount of downward solar radiation passing through the snow.

### 2.4. Ice Thickness Probe

**Figure 4:**
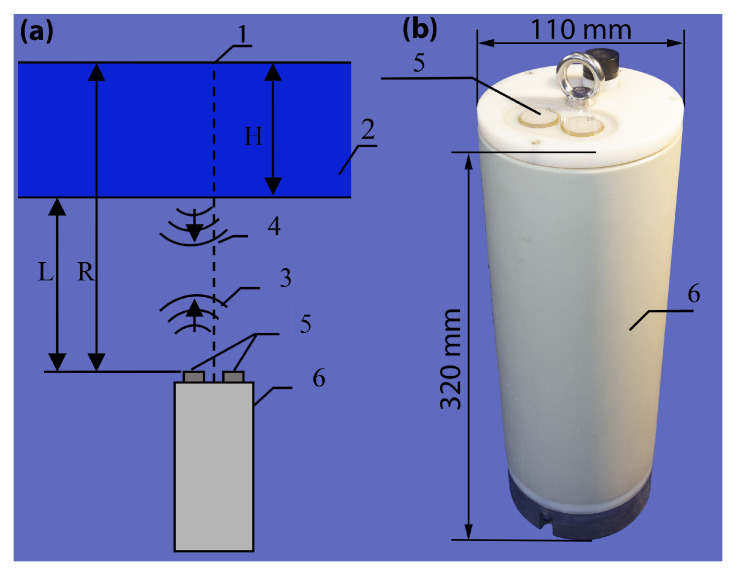
(**a**) Operating principle of the ice thickness meter: 1—suspension system, 2—ice, 3—emitted signal, 4—reflected signal, 5—hydroacoustic transducers, 6—device body; (**b**) photo of the device body.

An ultrasonic range finder based on the inverted echo sounder principle was developed to measure ice thickness. The measurement method was chosen to meet the requirements for frequent and highly accurate measurements, which would provide data on the daily dynamics of the ice thickness. For this purpose, emitting and receiving hydroacoustic transducers were built into the top cover of the recorder housing. The device installation concept is shown in Figure 4. The recorder unit is suspended on a tether in the water at a fixed distance, *R*, from the ice surface. The essence of the hydroacoustic method is that a modulated acoustic signal is emitted into the water, then reflected from the ice bottom and recorded by the receiver. The distance to the ice–water interface *L* and, hence, the ice thickness, *H*, can be calculated from the information on the time elapsed between transmission and reception and the sound speed in water (Figure 4).

To accurately determine the distance to the ice bottom, it is necessary to know the sound speed profile in the water. The propagation of acoustic waves in a medium is determined by its elastic properties. Liquids and gases do not have shear elasticity. On the other hand, when two adjacent layers come together or move apart, return forces occur in them, preventing shear and tensile deformations. As the vibrations of the particles of the medium occur in the direction of the returning elastic forces, the propagation of the initial displacement front is possible only in the form of a longitudinal wave. The following expression determines the velocity of longitudinal waves, *V*, in water:

(1) $$V=\sqrt{B/\rho },$$

where *B* is the bulk modulus of elasticity, ρ is the density of the medium. *B* and ρ depend on temperature *T*, salinity (or total dissolved solids, TDS) *S* and hydrostatic pressure *P*. This dependence has been established experimentally and is represented by various empirical equations, in particular, the widely known Chen–Millero formula for freshwater [28]. In our application, the Thermodynamic Equation of Seawater (TEOS-2010) is used to calculate the speed of sound [29], in which pressure dependence can be neglected due to the shallow installation depth of the recording unit. The average value of TDS in Lake Baikal is about 96 mg/L, and its total variations in the under-ice layer do not exceed 10 mg/L [30,31], which corresponds to negligibly small changes in sound velocity at 0.015 m/s. This will lead to a 0.02 mm deviation in the determination of the distance on a 1 meter base, which is less than the resolution of the instrument. The difference in pressure with deployment depth changes by 1 m also adds a comparable static error. The thermal expansion of the suspension cable has the strongest effect on the measurement of the distance to the lower boundary of the ice. As the thermal expansion coefficient of the stainless steel cable does not exceed $19\times 10^{-6}$ K${}^{-1}$ [32], its relative change with a 2 m suspension length and a range of the under-ice temperatures from 0 to +4 °C will be 0.155 mm. Therefore, taking into account the above-listed factors, the total uncertainty of the measurement of the distance to the lower boundary of the ice at the 2 m suspension length will not exceed 0.2 mm. Since we use high-precision temperature compensated crystal oscillator DSB321SDN (DAISHINKU Corp., Kakogawa, Hyogo, Japan) with a frequency tolerance of 1.5 ppm over a wide temperature range, the accuracy of time-of-flight measurements is so high that it can be neglected compared to other influencing factors. Thus, the vertical profile of sound velocity is calculated from the temperature profile measured with thermistor string with given average hydrostatic pressure and average under-ice water TDS for the studied water body.

#### 2.4.1. Transmitting and Receiving Circuit Design

A lead zirconium titanate-based piezoelectric ceramic transducer in the form of a 20 mm diameter disc with a 6 mm thickness and a resonance frequency of 360 kHz was used as a transmitting and receiving element. To emit a powerful prolonged phase-manipulated signal, it is necessary to drive the piezoelectric transducer with a high voltage. For this purpose, a DC-DC step-up converter from 12 V to 400 V based on the LTC3721 (Analog Devices, Norwood, MA, USA) chip was used in the transmitter circuit. The PWM signals from the converter are applied to the transformer primary winding, shortening it to the ground with transistors connected in a push-pull circuit. The feedback is organized on the TL431 chip via an optocoupler. The transmitter is designed with a full bridge circuit using N-channel transistors and high-power drivers, IRS21867 (International Rectifier). Each of the drivers receives two control signals from the main microcontroller to drive the upper and lower transistor, respectively. The power part of the MOSFET gate drivers is supplied by the galvanically isolated supply voltages generated on the additional secondary windings of the transformer. The transistors of the two drivers operate in anti-phase, alternately connecting the piezoelectric transducer to +400 V and to “ground”, thus the peak-to-peak range of the output signal is 800 V.

The receiver is based on a high-speed, low-noise two-stage OPA2350 amplifier. A digital potentiometer chip, MCP4551 (Microchip Technology Inc., Chandler, AZ, USA), is used to adjust the gain in the feedback of the second amplifier stage. To protect the amplifier from possible high voltage peaks on the piezoceramic, two oppositely directed diodes are placed at its input, so the limiting threshold of the input signal is about ±0.6 V. The amplified signal is fed to a high-speed 8-bit ADC ADS830 (Texas Instruments, Dallas, TX, USA) operating at 16 MHz. A 64KB IDT7208 (Integrated Device Technology, San Jose, CA, USA) high-speed asynchronous dual-port FIFO buffer is used to record the digitized data. Thus, the instrument directly records the waveform of the reflected signal, allowing its further post-processing.

#### 2.4.2. Principle of Operation

**Figure 5:**
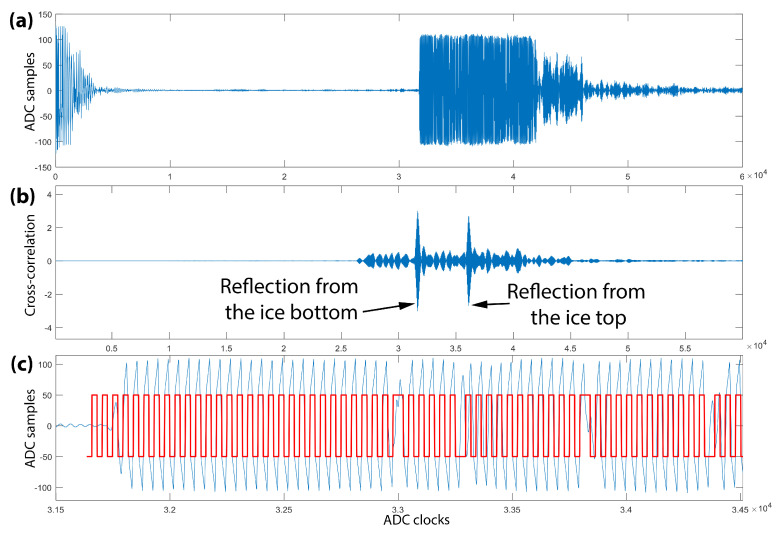
(**a**) Reflected signal waveform; (**b**) cross-correlation function of reflected signal with the reference signal; (**c**) zoomed in fragment of the reflected signal (blue line) with the superimposed reference signal (red line).

To increase the measurement accuracy of the time taken by an acoustic pulse to travel to the ice bottom and back, an M-sequence with a duration of 20 bits was applied as an emitting sound pulse. Such binary sequences have low autocorrelation sidelobe levels, so they improve the signal-to-noise ratio in radar applications [33]. The controller generates six periods of 330 kHz carrier frequency for each bit, changing the oscillation phase by 180 degrees when the bits are switched. The reception is performed by a high-speed ADC storing the echo waveform in a FIFO buffer. An example of the reflected waveform and its cross-correlation function with the reference signal is shown in Figure 5. The cross-correlation function demonstrates that two reflected signals are received (two peaks on it), while on the waveform it is impossible to distinguish them because of their overlap. The first receiving signal was reflected from the ice bottom, and the second signal passed through the ice and was reflected from the upper ice surface. The correlation method of detection distinguishes the difference in signal arrival time with the accuracy of one ADC cycle (62.5 ns), which corresponds to 0.05 mm in the distance resolution. The absolute precision of ice thickness measurements is attributed to the accuracy of the determination of the hanging system length during the installation of the instrument. If the length of the hanger was not initially known, it can be calculated later by the events of freezing of the temperature sensors in the ice. Therefore, the developed instrument can accurately determine the distances to the lower ice surface and provide information about the signal propagation time in the ice, which enables measuring the dependence of the average velocity of the sound in the ice on its temperature. The working range of distance measurements from the instrument to the ice bottom is 0.2 to 2.8 m by default. The maximum range can be increased up to several times by repeatedly filling the FIFO buffer.

### 2.5. Snow Thickness Meter

**Figure 6:**
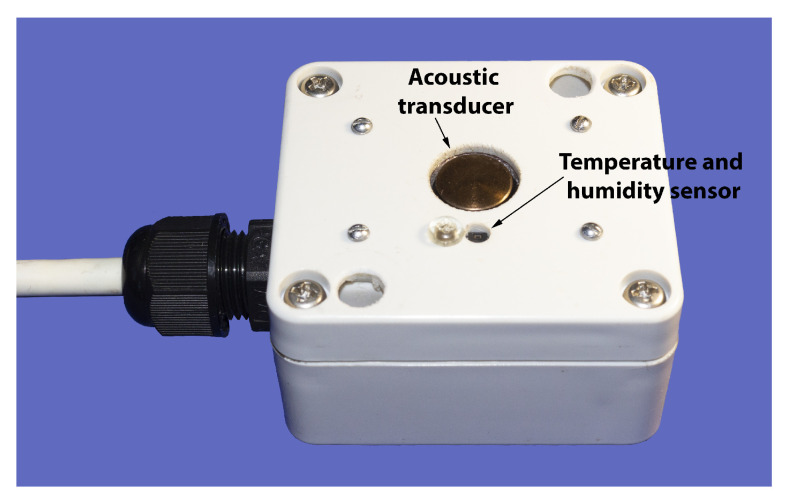
Snow thickness meter with integrated temperature and humidity sensor.

An ultrasonic range finder similar to the underwater one was developed to measure snow thickness. The sensor is positioned in the air at a fixed distance from the ice surface and measures the transit time of the acoustic signal to and from the upper boundary of the snow. During precipitation, the reflection boundary shifts towards the sensor, thereby reducing the time between transmission and reception. To improve measurement accuracy, temperature and humidity sensors were integrated into it to make corrections to the sound velocity in the air depending on these parameters. The carrier frequency of the emitted signal is 40 kHz. The developed sensor has a distance resolution of about 1 mm and an accuracy of 1 cm. A photo of the sensor is shown in Figure 6.

## 3. Results of the First Field Experiment

**Figure 7:**
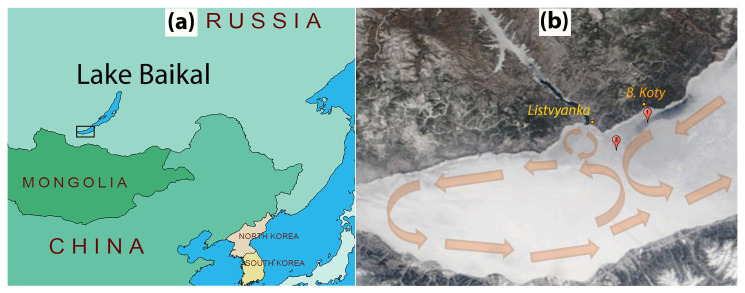
(**a**) Geographical location of the study site; (**b**) Ice conditions at the end of the experiment (on 31 March 2014) with the pattern of mean currents according to [34] in the southern part of Lake Baikal and locations of the autonomous measurement stations. The satellite image (Terra MODIS true color band composition) obtained from the Irkutsk Center of Remote Sensing [35]. Note the stronger ice melt in the area of the jet current around Station 1 visible as a dark area in Panel (**b**).

The first test run of the ASLIM was conducted in 2014 in the southern part of Lake Baikal (Figure 7a). Lake Baikal, the largest lake by volume on Earth, most closely resembles the Arctic Ocean in terms of seasonal ice dynamics. Thanks to the strong winter cooling under the influence of the Siberian atmospheric pressure maximum, Lake Baikal shows a steady ice cover over the entire lake for three to five months of the year. Two measuring systems along with current velocity meters were installed at different sites, after a stable ice formation, on 13 February 2014 (Figure 7b).

**Figure 8:**
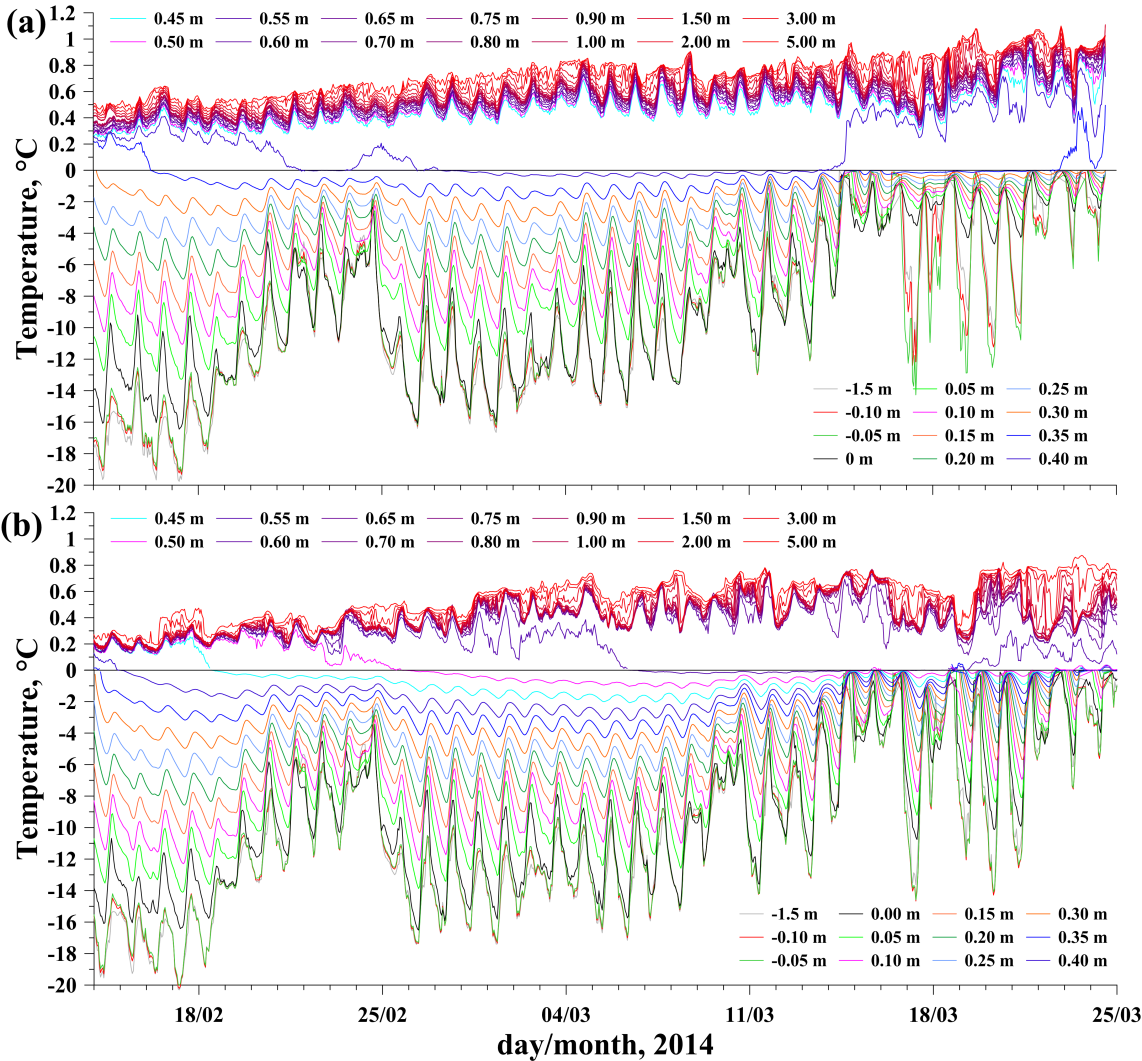
Temperatures in the ice, water and air at Station 1 (**a**) and Station 2 (**b**). The legends show the distance from sensors to the ice surface, positive direction — downward. Intersections of temperature curves with the abscissa (0 °C) correspond to the times of sensor freezing in the ice. Note the different scales for positive and negative temperatures.

**Figure 9:**
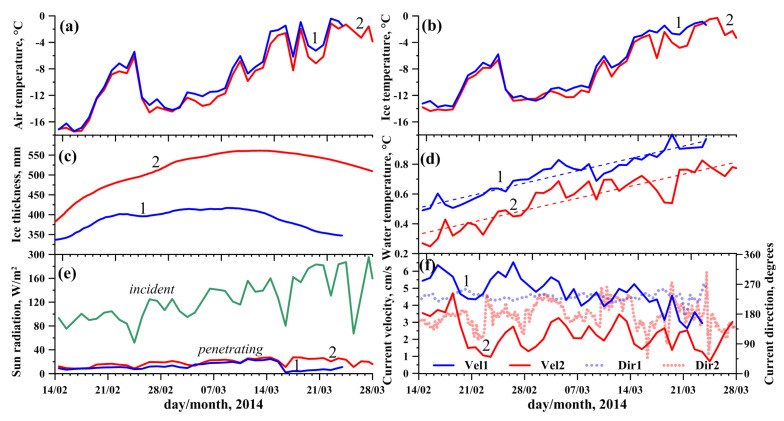
Daily averaged data on the Lake Baikal ice regime during the observations for Station 1 (blue lines) and Station 2 (red lines): (**a**) air temperatures at 1.5 m above the ice, (**b**) temperatures of the ice surface, (**c**) ice thickness, (**d**) water temperatures at 5 m depth and calculated linear trends, (**e**) incident solar radiation and penetrating water (at 1.5 m depth) and (**f**) current velocities and directions at a 1.5 m depth.

Station 1 was installed in the area of the Bolshiye Koty settlement, at the site with increased under-ice currents with the following coordinates: 51°51.850${}^{\prime }$ N 105°04.711${}^{\prime }$ E; the initial ice thickness was 34 cm. An INFINITY-EM two-dimensional electromagnetic current meter (JFE Advantech Co., Ltd., Nishinomiya, Japan) was used to measure the current velocities (velocity range ±5 m/s, resolution 0.02 cm/s and accuracy ±1 cm/s). Station 2 was installed at a distance of 13 km NW closer to the Listvyanka settlement, at the site with the following coordinates: 51°47.498${}^{\prime }$ N 104°56.639${}^{\prime }$ E; the initial ice thickness was 38 cm. The station was equipped with a Doppler current velocity meter, Sontek MicroADV (SonTek—A Xylem brand, San Diego, CA, USA), operation frequency 16 MHz, programmed velocity range from 3 to 250 cm/s, resolution 0.01 cm/s, accuracy 1% of measured velocity or 0.25 cm/s. Both current meters were deployed at a distance of 1.5 m beneath the ice surface. Installation of the instruments allowed us to obtain continuous data on temperature in the ice and under-ice water (Figure 8 and Figure 9b,d), incoming and penetrating solar radiation (Figure 9e), ice thickness variations (Figure 9c) and direction and velocity of under-ice currents (Figure 9f).

The variations in air temperatures and incident solar radiation on both stations were rather similar (Figure 9a,e). After installing the instruments, the ice was free from snow and ice surface temperatures at both stations were nearly equal, closely following the air temperatures at a height of 1.5 m (Figure 9b). During the entire measurement period, an almost constant portion of the incoming solar radiation in the range of 10 to 15% penetrated the ice at both stations (Figure 9e). Therefore, steady warming of under-ice water caused by volumetric absorption of penetrating solar radiation began immediately after the installation of the instruments (Figure 8 and Figure 9d). The average warming rate was 0.012 and 0.011 °C per day at Stations 1 and 2, respectively. More uniform warming of water at Station 1 (R${}^{2}$ = 0.9) was due to the constant inflow of warm water (see current directions in Figure 9f) from the less snow-covered northeastern part of southern Baikal (Figure 7b). Station 2 was under the influence of two minor circulation cells of under-ice currents [34], and a greater irregularity of current directions (Figure 9f) and, as a consequence, of water warming (R${}^{2}$ = 0.87), was observed there (Figure 9d) due to periodic intrusions of cold water from the snowier and less warmed western part of southern Baikal (Figure 7b).

After a large snowfall on March 16, a stable snow cover was formed at Station 1, leading to a decrease in the fraction of penetrating solar radiation to 5% and an increase in the temperature of the ice surface due to its thermal insulation from the air (Figure 9b,e). Despite the very close weather conditions at the stations, the dynamics of ice thickness there were dramatically different (Figure 9c). At Station 1, the ice cover stopped growing the week after the deployment, with the arrival of warm weather on February 21 to 24, so the sensor located at a 0.4 m depth melted out of the ice (Figure 8a). Subsequent cooling did not cause active growth of ice thickness, which increased only by several millimeters in two weeks, and continuously decreased thereafter, when general warming caused active ice ablation.

In contrast to Station 1, the ice at Station 2 grew until mid-March, with only a slight slowdown during the February warming (Figure 9c). Therefore, by the end of the observations, there was a significant difference of more than 15 cm between the ice thicknesses at the stations. The different ice cover development of the two stations can apparently be attributed to the differences in the local heat budget, which are analyzed below.

### Heat Balance at the Ice–Water Interface

The heat balance at the ice–water interface (IWI) can be expressed as follows [36]:

(2) $$Q_{cw}=Q_{ci}-Q_{L},$$

where the vertical coordinate is directed downwards with origin *z* at the top of the ice; $Q_{cw}$ is the conductive heat flux from/to the water,

(3) $$Q_{cw}=C_{pw}\rho_{w}\kappa_{w}{\left.\frac{dT}{dz}\right|}_{z=h_{i}+0},$$

$Q_{ci}$ is the conductive heat flux from/to the ice,

(4) $$Q_{ci}=C_{pi}\rho_{i}\kappa_{i}{\left.\frac{dT}{dz}\right|}_{z=h_{i}-0},$$

and $Q_{L}$ is latent heat release/consumption due to the phase change (freezing or melting),

(5) $$Q_{L}=\rho_{i}L_{f}\frac{dh_{i}}{dt},$$

$h_{i}$ is the ice bottom coordinate, and $dh_{i}dt^{-1}$ is the rate of basal ice melting/growth; $\rho_{i}L_{f}$ is the product of the ice density and latent heat of fusion.

Temperature at the IWI is fixed at the melting point of 0 °C that corresponds to the following thermodynamic characteristics: molecular heat diffusion coefficient for fresh water is $\kappa_{w}\approx 1.4\times 10^{-7}$ m${}^{2}$ s${}^{-1}$, molecular heat diffusion coefficient for ice is $\kappa_{i}\approx 1.1\times 10^{-6}$ m${}^{2}$ s${}^{-1}$, the product of the water heat capacity and density is $C_{pw}\rho_{w}\approx 4.18\times 10^{6}$ J K${}^{-1}$ m${}^{-3}$ and the same product for ice is $C_{pi}\rho_{i}\approx 1.96\times 10^{6}$ J K${}^{-1}$ m${}^{-3}$ [37].

**Figure 10:**
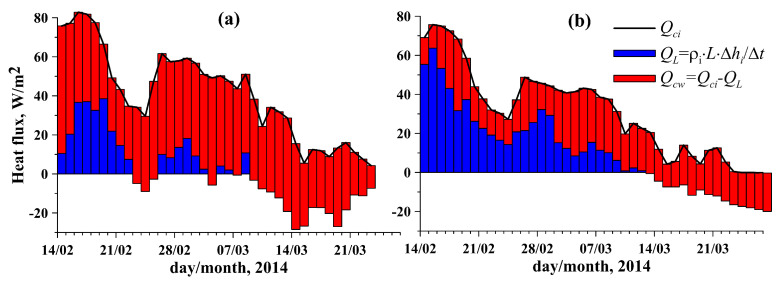
Daily averaged heat balance at (**a**) Station 1 and (**b**) Station 2.

Equation (2) can be applied for reliable estimation of the ice–water heat flux, $Q_{cw}$ from the temperature profile within the ice cover and the time variations in the ice thickness, $dh_{i}dt^{-1}$, both measured by the ASLIM with high temporal and spatial resolution [25]. Calculation of the heat flux within the ice was carried out from temperature readings of the sensors located at 10 to 15 cm from the ice bottom. The resulting daily averaged heat balances at the IWI calculated from Equations (2), (4) and (5) for both stations are shown in Figure 10. The heat fluxes in the ice were rather close for both stations, whereas the heat flux from water to ice differed significantly. The ice–water heat flux was generally stronger at Station 1, correlating with more intense currents observed there (Figure 9f). Already at the beginning of the observations period, in February, upward conductive heat loss, $Q_{ci}$, of up to 80 W m${}^{-2}$ was compensated to 30–80% by the heat supply from the water column, $Q_{cw}$. The latter significantly reduced the ice growth rate and latent heat release (cf. red and blue areas in Figure 10).

**Figure 11:**
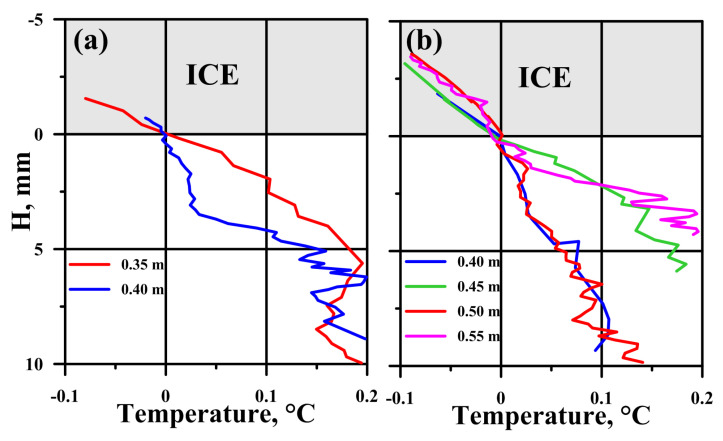
Fine-scale temperature profiles at Station 1 (**a**) and Station 2 (**b**) obtained during the freezing of miniature analog sensors in the ice with the one-hour averaging. H—distance from the ice–water interface.

As the ice thickness grows, the lower ice boundary gradually approaches the sensors originally located in the water, which is equivalent to the motion of sensors toward the ice–water interface. Based on the changes in ice thickness with high resolution (Figure 9c), it is possible to convert the temperature changes versus the time (Figure 8) to the temperature versus distance from the ice bottom (Figure 11) by coordinate transformation from time to vertical coordinate with moving origin.

**Figure 12:**
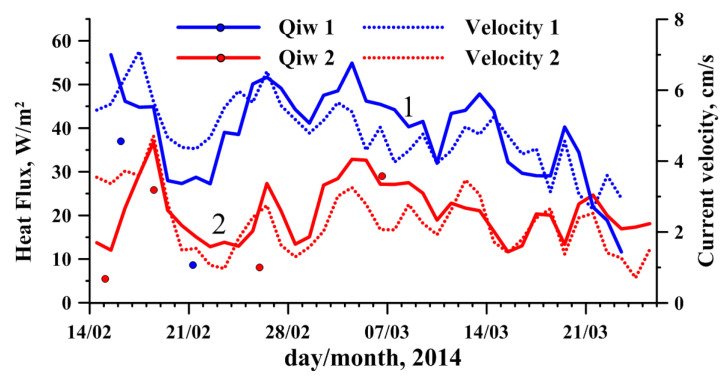
Daily averaged heat fluxes at the ice–water interface (solid lines) calculated from heat balance Equations (2), (4) and (5) and current velocities (dashed lines) at Station 1 (blue lines) and Station 2 (red lines); the circles are instantaneous values of heat fluxes determined by the temperature gradient in the thin under-ice water layer when thermal sensors froze in the ice (3).

Evidently, before freezing into the ice, the sensors moved from a turbulent water mass with large temperature fluctuations and passed through the laminar layer, with an almost linear change in temperature. Based on the obtained temperature profiles, we can estimate the thickness of the laminar layer, which ranged from 2 to 3 mm. Based on the temperature gradient in the laminar layer, we can estimate the heat flux from the water according to Equation (3). The results of flux calculations are shown in Figure 12 together with daily averaged values calculated from the heat balance and current velocities at both stations.

It can be seen that the heat fluxes obtained by different methods are close to each other and correlate well with the intensity of currents under the ice.

## 4. Discussion

Until recently, ice cover measurements were confined to mostly irregular human-made binary observations on ice cover presence/absence. Nowadays, continuous monitoring of the ice cover belongs to key tasks of modern climate research, providing actual information on the climate change in cold regions and on the frequency of extremely warm or extremely cold winters. A strong advance in ice monitoring worldwide has been provided by the recent development of remote sensing methods, which yield reliable large-scale data on ice cover duration and spatial extent. However, remote sensing has only limited ability to measure the ice thickness, thereby failing to reliably estimate the ice mass budget. Moreover, a correct interpretation of the ice observations requires coupling of the ice mass budget to the heat budget of the water–ice–atmosphere system, which is the main driver of the ice formation and melt. Therefore, a demand exists for in situ platforms capable of providing these data. Such platforms have to meet several requirements ensuring autonomous work in the harsh environmental conditions. The trade-off between the high temporal resolution of measurements and the up to several months of autonomous deployment put strict limitations on the power consumption. A high resolution and accuracy of the sensors are needed to register the variability in the major measured environmental variables—temperature and solar radiation—in low-energy winter conditions. Additional difficulties are introduced by low temperatures and potential icing of the platform.

We developed original equipment recording hydrometeorological parameters in the atmosphere, ice and water with a high spatial and temporal resolution to study the vertical heat and mass exchange in the water–ice system. The first trial installation of two instances of the measuring systems provided detailed information about the processes accompanying the growth and melt of the ice cover on Lake Baikal. The obtained data allowed us to determine the ice heat balance components with good accuracy. The fine-scale temperature profiles derived near the ice–water interface enabled us to determine the dimensions of the laminar boundary layer and provide additional estimates of heat fluxes from water to ice consistent with the values obtained by the heat balance method.

In this study, we recorded extremely high heat flux values from water to ice (more than 50 W m${}^{-2}$) during the period of ice growth. The fluxes are likely related to the water circulation pattern beneath the ice. Despite the thin snow cover, the convection due to penetrating solar radiation cannot produce such a strong boundary mixing, exceeding the fluxes measured in small lakes [38,39] up to an order of magnitude. The heat fluxes at Station 1 were so strong that two weeks after the retrieving of the monitoring systems, on April 9, there was already open water of several kilometers in size, whereas the entire southern Baikal was free of ice a month later, only by May 11.

Thereafter, from January to April 2016, we carried out synchronous observations using three developed ice monitoring systems for the comprehensive study of the influence of contrasting (in terms of snow thickness and of the under-ice current velocities) conditions on the formation of heat flux at the ice–water interface [26]. A mathematical model of vertical heat transfer in a two-layer system with phase transition (Stefan problem) was developed to interpret the results of in situ measurements. As a result of solving the inverse ice growth problem, the coefficients of effective thermal diffusivity in the under-ice water were calculated, and the vertical distribution of heat fluxes in the water–ice system was estimated. The heat fluxes calculated using the developed model served as additional confirmation of their reliable determination by the heat balance method. The results of this experiment also showed that there are areas in Lake Baikal where the heat fluxes are comparable to the values in small lakes (<5 W m${}^{-2}$) [26].

The next year, we carried out a special field experiment to closely estimate the effects of under-ice current intensity on the ice–water heat flux under the same meteorological conditions [40], combining high temporal and spatial resolution temperature measurements with the developed systems and fine-scale registration of current velocities under the ice base with a Doppler current velocity profiler, HR Aquadopp (Nortek AS, Norway). The data on velocity fluctuations provided information on the variability of mean currents under the ice as well as on the characteristics of turbulent mixing in the ice–water boundary layer in the form of the dissipation rate of the turbulent kinetic energy (TKE). The main motivation of the study was to seek the relationship between the shear mixing and stratification, on one hand, and the heat release from water to the ice cover, on the other hand. To explain stratification effects, we proposed a model of the turbulent energy budget based on the length scale incorporating the dissipation rate and the buoyancy frequency (Dougherty–Ozmidov scaling). The model agrees well with the observations and yields a scaling relationship for the ice–water heat flux as a function of the shear velocity squared [40].

These studies have revealed that in different years and different areas of Lake Baikal the heat flux from water to ice varies within a wide range (from 2 to 50 W m${}^{-2}$), constituting from 5 to 100 percent or more of the heat flux in the ice. This result demonstrates the significant role of underwater circulation in the formation of heat flux at the ice–water interface and, as a consequence, the ice thickness in such large water bodies as Lake Baikal. Therefore, the shear instability generated by the currents can be considered the main winter driving force of heat transport from the water column to the ice base because the contribution of the heat flux due to convection driven by salt release from the growing ice in Baikal does not exceed 1 W m${}^{-2}$ [30], and the intensity of turbulent exchange due to volumetric absorption of solar radiation becomes significant only by the spring [41,42].

In 2018, one of the measurement systems was used to continue the previous studies [38,43] on the small Arctic Lake Kilpisjarvi, the article about which is being prepared. Unlike Lake Baikal, from January to late April, the heat fluxes from water to ice did not exceed 4 W m${}^{-2}$ in this lake.

Our monitoring platform implements modern advances in the environmental sensing to obtain a reliable autonomous solution suitable for application in a wide range of ice-covered conditions. Originally designed for freshwater lakes, the configuration of the sensing platform reflects specific features of lake ice cover: high transparency of the freshwater ice and, as a result, the critical importance of short-wave solar radiation for the total heat budget of the ice and underlying water [44], relatively low flow velocities at the ice base resulting in weak water–ice heat fluxes and requiring high vertical resolution of the temperature gradients in the vicinity of the ice–water interface. However, the modular concept of the proposed platform ensures its flexibility and adjustability to different ice-covered systems, including (sub-)polar seas and coastal and estuarine waters.

In our device we used a sensor spacing of 5 cm, which is higher than that of SIMBA buoys, where temperature sensors are distributed every 2 cm. Results of our studies together with the mathematical modeling have revealed that the 5 cm step between the sensors is sufficient to verify ice thermodynamic models. Using the typical values of the ice thermal conductivity (2 W m${}^{-1}$ K${}^{-1}$) and specific heat (2 kJ kg${}^{-1}$ K${}^{-1}$), the spatial resolution of 5 cm allows for resolving temporal scales of ∼40 min, which is sufficient to effectively capture (sub-)diurnal variations. An increase in vertical resolution in our design is technically possible (e.g., a 2 cm resolution will reduce the captured temporal scales to ∼7 min) and may appear reasonable for waters with relatively thin ice cover. The trade-off in this case consists in the increase in the linear dimensions of the construction with potential disturbing influence on the measured temperatures and loss of robustness. Therefore, in our device, we focused on minimizing the linear dimensions of temperature sensors themselves and ensuring that the sensor tips are as far away from the main rod of the measuring system and wooden support frame as possible. This allows us to measure the in situ temperature, without additional distortion by the influence of the temperature transfer from the load-bearing structure itself. An advantage of ASLIM is the high sampling rate of every 1–2 min, whereas all other IMB buoys provide measurements once an hour. The sub-hourly sampling is particularly critical for capturing the temperature variations in the ice-adjacent water layer.

Another a distinctive feature of ASLIM is the high resolution of the ice thickness meter of 0.05 mm, which allows for resolving the fine-scale processes occurring in low-flow environments, and measuring the ice growth every 2–4 min at the initial stage of its formation. For comparison, the resolution of rangefinders on SIMB-3 and TUT buoys are 1 cm [13], and typical accuracy of the ice thickness retrieved through the sea ice temperature profile measurements from SIMBA buoys using automatic discrimination algorithms is in the range of 20–90 mm [18] or 10–90 mm in another study [17].

An important difference between our system and commercially available buoys is the presence of several light sensors in the air, in the ice and in the water which allows for estimating the attenuation of solar radiation in the ice and snow covers and in the water column. In this regard, the experimental development of a chain of 48 side-ward viewing multispectral irradiance sensors at 5 cm vertical spacing [45] is of particular interest. Its first deployment showed that the sensor chain successfully captures the spatiotemporal variation of the in-ice light field from the surface through the ice to the underlying water.

Currently, four units of the ASLIM have been assembled and more are under development. The monitoring systems can be used both for long-term observations of the ice cover dynamics in different climatic conditions and for development of and verifying ice models, with their subsequent integration into the climate modeling and weather prediction systems. Another direction of ASLIM use consists in investigation of the spatial heterogeneities in the ice cover characteristics in Lake Baikal, extendable to other large ice-covered lakes and marginal seas. Additionally, it would be interesting to investigate with several systems the areas of appearance of giant ring structures on the Baikal ice cover [46,47]. Apart from Lake Baikal, the system has been deployed and tested in small polar lakes and in ice-covered rivers. In particular, a test deployment in the Selenga River in 2021 showed a good potential of the system to obtain reliable data even at strong flow velocities up to 50 cm/s, when provided a rigid hanger of the main body with transducers to the ice cover. Herewith, a simplified version of the system can be potentially used for automatic ice cover monitoring by governmental and public agencies in applications related to on-ice transportation and other critical safety issues.

One potential extension of the monitoring platform consists in parallel monitoring of weather parameters: wind, air humidity and long-wave radiation balance between ice surface and the atmosphere. It will allow estimation of the total heat and mass budget of lake ice cover including the ice–atmosphere boundary layer [48,49] and contribute to improvement of the fully coupled land–atmosphere modeling [50]. Including the weather variables in the monitoring is especially important for remote sites with limited availability of regular weather observations. Size and energy consumption are the two major factors limiting the extension of the platform with new sensors. Potential inclusion of electric conductivity sensors can provide estimation of the water/ice TDS, as a factor affecting the vertical heat transport and the melting point of the ice. Such an extension would provide a better insight in the ice cover dynamics of marine and brackish environments, but would, however, require stable, robust and accurate conductivity sensors suitable for long-term autonomous deployment.

## 5. Conclusions

We have developed an autonomous system providing in situ continuous monitoring of vertical temperature distribution in the air–ice–water system for several months with simultaneous records of solar radiation incoming at the lake surface and passing through the snow and ice as well as of snow and ice thicknesses. The use of modern electronic components and miniature analog and digital sensors enabled us to create a spatially distributed set of addressable sensors operating on a common four-wire bus. This approach allows for flexibly expanding the number of sensors in the measurement system and freely planning their spatial arrangement. The system was tested in several studies on Lake Baikal and demonstrated high reliability in deriving the ice heat balance components during the ice-covered period.

## Data Availability

Data are available online through the following link: https://hlserver.lin.irk.ru/shs/icemon/ (accessed on 16 December 2021).
